# Neuroprotective Effects of Fluoxetine Against Chronic Stress-Induced Neural Inflammation and Apoptosis: Involvement of the p38 Activity

**DOI:** 10.3389/fphys.2020.00351

**Published:** 2020-05-11

**Authors:** Yuxiao Zhao, Pan Shang, Meijian Wang, Min Xie, Jian Liu

**Affiliations:** ^1^Queen Mary School, Nanchang University, Nanchang, China; ^2^Department of Endocrinology, The Second Hospital of Shandong University, Jinan, China; ^3^Department of Emergency Medicine, The First Affiliated Hospital of Nanchang University, Nanchang, China

**Keywords:** neuroprotection, fluoxetine, neuroinflammation, apoptosis, p38, depression

## Abstract

Depression is considered a widespread neuropsychiatric disease associated with neuronal injury within specific brain regions. Fluoxetine, a selective serotonin reuptake inhibitor, has been widely used in depressed patients. Recently, fluoxetine has demonstrated neuroprotective effects apart from the effect on serotonin. However, the underlying mechanism involved in this neuroprotection remains unclear, in particular, whether fluoxetine exerts antidepressant effects via protecting against neuronal injury. Here, we found that treatment with fluoxetine (10 mg/kg, i.p.) for 2 weeks ameliorated depression-like behaviors in a chronic unpredictable mild stress (CUMS)-induced rat model of depression and was accompanied with an alleviation of glia activation and inhibition of interleukin-1β (IL-1β), interferon gamma (IFN-γ), and tumor necrosis factor-α (TNF-α) expression in the hippocampal dentate gyrus (DG) region. Meanwhile, CUMS rats treated with fluoxetine showed reductions in neuronal apoptosis and a downregulation of the apoptotic protein Bax, cleaved caspase 3, and caspase 9 levels. These effects appear to involve a downregulation of p38 mitogen-activated protein kinase (MAPK) signaling within the DG hippocampus as the specific inhibitor of p38 MAPK, SB203580, significantly suppressed apoptosis, as well as ameliorated depressive behaviors resulting from CUMS exposure. Moreover, fluoxetine could rescue neuronal deterioration and depression-like phenotypes caused by overexpression of p38 in DG. This finding extends our knowledge on the antidepressant-like effects of fluoxetine, which appear to at least partially profit from neuroprotection against inflammation and neuronal apoptosis via downregulation of the p38 MAPK pathway. The neuroprotective mechanisms of fluoxetine may provide some novel therapeutic avenues for stress-related neurological diseases.

## Graphical Abstract

Schematic depicting fluoxetine protect against chronic stress-induced neural inflammation and apoptosis through targeting p38 signaling.

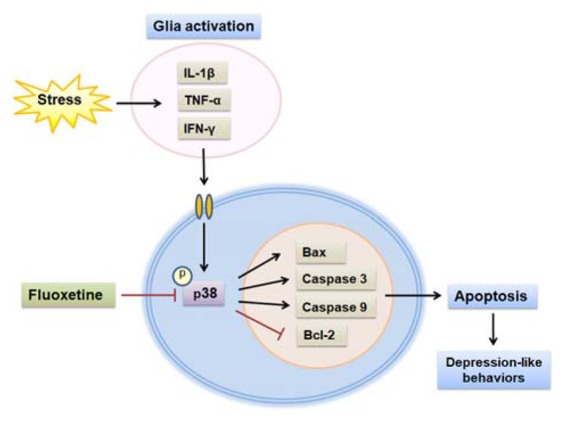

## Introduction

Neuroinflammation within specific brain regions is considered one of the critical risk factors responsible for the pathogenesis of depression (Alcocer-Gomez et al., 2015; Dean and Keshavan, 2017; Adzic et al., 2018). Accumulating evidence has indicated that several pro-inflammatory cytokines including interleukin-1β (IL-1β), tumor necrosis factor-α (TNF-α), and interleukin-6 (IL-6) were all increased in patients with major depressive disorder (MDD) (Schiepers et al., 2005; Song et al., 2009; Hannestad et al., 2011). This array of factors then contributes directly to the diverse forms of neuronal injury associated with this condition (Rothwell, 2003). However, details regarding the underlying mechanisms of these pathophysiological processes in depression are not fully understood. Thus, seeking potential therapeutic targets and corresponding effective strategies against inflammation-induced neuronal injury will be crucial for new and more effective treatments of depression.

Fluoxetine, a classical selective serotonin reuptake inhibitor (SSRI), is widely used as a psychotherapeutic drug in the treatment of depression (Bastos et al., 2015). Apart from its effects on serotonin, fluoxetine has been demonstrated to play a crucial role in anti-inflammatory, anti-tumor, and neuroprotective effects (Ghosh et al., 2015; Ng et al., 2015). Accordingly, fluoxetine can also mitigate neurological deficits by inhibiting acute ischemia/reperfusion-induced neuronal injury and inflammation-induced neuronal apoptosis (Shan et al., 2016). Such effects of fluoxetine may then also be involved with suppressing inflammation-induced neuronal death involved with depression and suggests a new therapeutic mechanism involved with this treatment. However, the exact mechanisms for the capacity of fluoxetine to inhibit neuroinflammation after chronic stress are not clearly understood. The identification of molecular pathways involved in this process would greatly aid in providing novel therapeutic and preventative targets in the treatment of this depressive disorder.

In the present study, we first investigated the neuroprotective effects of fluoxetine treatment in the chronic unpredictable mild stress (CUMS)-induced depression, and found that fluoxetine could significantly suppress neuroinflammation and apoptosis and ameliorated the depression-like behaviors in rats through p38 mitogen-activated protein kinase (MAPK) pathway within the dentate gyrus (DG) of hippocampus.

## Materials and Methods

### Animals

Male Wistar rats weighing 160–180 g were obtained from the Experimental Animal Centre of Nanchang University. All animal experimental procedures were performed according to the guidelines of the ethics committee of the Medical Department of Nanchang University and the International Guiding Principles for Animal Research provided by the International Organizations of Medical Sciences Council (CIOMS). All efforts were made to reduce the animals’ suffering.

### Animal Model of Depression

The CUMS procedure with minor modifications was used to generate an animal model of depression (Mao et al., 2009). Briefly, rats were housed individually in a colony room, and a stressor was applied daily to each rat in a random order and at unpredictable time-points for a 5-week period. The stress regime included cold swimming (5 min, 4°C), cage shaking (2 h), 24 h of food deprivation followed by 24 h of water deprivation, physical restraint (2 h), overnight illumination, foot-shock (0.5 mA, 0.5 s), and wet bedding (24 h).

### Drug Treatments

The dose and route of fluoxetine (Sigma-Aldrich, United States) administration is based upon a previous study (Lapmanee et al., 2013). Fluoxetine was administered via an intraperitoneal (i.p.) injection for 2 weeks after CUMS procedure. The p38 MAPK antagonist SB203580 or DMSO was administered intracerebroventricularly (i.c.v.) daily at 60 min prior to stress stimulator over the 5-week CUMS period. The dose and route of SB203580 administration is based upon a previous study (Zheng et al., 2004). Rats were randomly allocated to one of the following groups (*N* = 18/group): (1) control (non-stressed), (2) CUMS, (3) CUMS treated with fluoxetine (10 mg/kg; CUMS + FLX), (4) CUMS pretreated with SB203580 (5 μg/kg; SB + CUMS), and (5) CUMS pretreated with DMSO (1.0 μl; DMSO + CUMS). The experimental schedule is presented in Supplementary Figure 1.

### Intracerebroventricular Injection

Rats were anesthetized with 2.5% isoflurane and placed in the stereotaxic apparatus. A portion of the parietal skull was then removed, and a guide cannula was inserted into the right lateral ventricle (coordinates from bregma: −1.5 mm; medial/lateral: ±1.0 mm; dorsal/ventral: −3.2 mm). After recovery from surgery, 10 μl of either SB203580 (0.1 μg/μl) or DMSO (0.1%, 1.0 μl) were micro-infused at a flow rate of 0.5 μl/min into the lateral ventricle daily at 60 min prior to CUMS procedures.

### Stereotaxic Injection of the AAV Virus

The AAV9–CMV–eGFP–p38 virus was constructed to overexpress p38 protein levels in the DG region. Rats were allocated to one of the following groups (*N* = 18/group): (a) AAV–eGFP, (b) AAV–p38, (c) AAV–p38 + fluoxetine. Purified AAV virus (∼10^12^ infection units per ml, 1–1.5 μl) were infused bilaterally into DG regions (from bregma: AP, −3.24 mm; ML, ±0.5 mm; DV, −4.8 mm) at a rate of 150 nl/min. The following assays were performed at least of 14 days after viral injection. The experimental schedule is presented in Supplementary Figure 1.

### Behavioral Tests

#### Forced Swim Test

Twenty-four hours post CUMS procedure, the forced swim test (FST) was performed to assess despair behavior in rats (Porsolt et al., 1977; Duman et al., 2007). Briefly, rats were placed individually in a cylinder of water (height: 80 cm, diameter: 30 cm, temperature: 25°C) for 15 min of forced swim training. Twenty-four hours later, each rat was placed in the cylinder for a 5-min test. The durations of immobility (floating with only limited movements to maintain their head above water) and swimming were recorded by an observer blinded as to the treatment group.

#### Sucrose Preference Test

The sucrose preference test (SPT) was used to evaluate anhedonia in rats (Mao et al., 2009). Briefly, after the adaptation session, rats were deprived of food and water for 24 h and then permitted free access to two bottles for a 3-h test, one containing 100 ml of sucrose solution and the other containing 100 ml of tap water. The sucrose preference was presented as: sucrose consumption/(water consumption + sucrose consumption) × 100%.

#### Open Field Test

The open field test (OFT) was used to measure the spontaneous exploratory behavior in rats as described previously (Walsh and Cummins, 1976). Briefly, rats were individually placed in the center of a square plywood platform (100 cm × 100 cm × 40 cm) and were permitted to explore freely for a 5-min session. The number of horizontal locomotor (segments crossed with four limbs) and exploratory activities (rearing and standing on the hind limbs) were recorded.

### Immunofluorescence Staining

One day after behavioral tests, six rats from each group were anesthetized and transcardial perfused with 4% paraformaldehyde (PFA). Brains were removed and post-fixed in PFA overnight at 4°C followed by a graded dehydration (10, 20, and 30% sucrose solution). Brain samples were cut into serial coronal frozen slices (30 μm) by oscillating blade microtome (Leica VT1200). The slices that contain DG regions were blocked in 5% BSA for 2 h and then incubated overnight with the primary antibodies anti-ionized calcium-binding adaptor molecule-1 (Iba-1) (1:500; WAKO, Japan), anti-glial fibrillary acidic protein (GFAP; 1:100; Proteintech, United States), anti-active caspase 3 (1:300; Abcam, United States), or anti-NeuN (1:400, Cell Signaling Technology, United States) followed by the appropriate fluorescent-conjugated secondary antibody (1:200; Sigma-Aldrich) for 1 h. Slides were then incubated with 4′,6-diamidino-2-phenylindole dihydrochloride (DAPI) at room temperature for 7 min. Images were captured using a scanning laser confocal microscope (LSM780; Carl Zeiss, Germany). For analysis, we focus on the hippocampal DG regions according to its coordinates (from bregma: AP, −3.20 mm ∼−3.60 mm; ML, ±0.5 mm; DV, −4.8 mm). The number of immune-positive cells was calculated as the mean of the numbers obtained from six pictures from each rat. If fluorescence intensity quantification is necessary, 20× confocal images of fluorescence staining slides and the negative control slides of DG were acquired using identical settings and further analyzed using Image-Pro Plus. The integrated optical density (IOD) of IHC signals was divided by area value of DAPI signals to derive the fluorescence signal intensity. Fluorescence intensities were expressed as a percent of control group. A minimum of six to eight images were selected from each rat for analysis by Image-Pro plus 6.0 software. The statistical results of quantifying fluorescence intensity were expressed as percentage ratios relative to the control group.

### Transmission Electron Microscopy

Six rats from each group were anesthetized with sodium pentobarbital (150 mg/kg, i.p.) 1 day after behavioral testing. The brain was quickly removed, and the DG hippocampus (1 mm × 1 mm × 1 mm) was dissected and placed in 2.5% glutaraldehyde at 4°C for 4 h. The tissue was then post-fixed with 1% OsO4 in 0.1 M PBS (pH 7.4) for 2 h at room temperature followed by dehydration and infiltration. Tissues were then embedded in resin and cut into ultrathin sections (70 nm) with an ultramicrotome. Sections were stained with 4% uranyl acetate for 20 min and with 0.5% lead citrate for 15 min and then examined using transmission electron microscopy (Philips Tecnai 20 U-Twin, Holland). In the present study, a minimum of 30 micrographs were randomly selected from each rat for analysis using ImageJ analysis software (NIH, Scion Corporation, Frederick, MD, United States).

### Western Blot Analysis

One day after behavioral testing, six rats per group were anesthetized with sodium pentobarbital (150 mg/kg, i.p.) and quickly decapitated for western blot analysis. Briefly, DG tissue was homogenized in ice-cold RIPA lysis buffer with a cocktail of protease/phosphatase inhibitors. The homogenate was centrifuged at 14,000 × *g* for 10 min at 4°C, and supernatants were collected. Protein concentrations in DG hippocampus was determined using BCA Protein Assay kits (Beyotime, China). Equal amounts of protein (30 μg) was loaded onto 8–15% SDS-PAGE gels for electrophoretic separation, then transferred onto PVDF membranes. Membranes were blocked in 5% non-fat milk for 1 h and probed with the following primary antibodies: Iba-1 antibody (1:1,000; WAKO, Japan), GFAP antibody (1:1,000; Proteintech, United States), anti-caspase-9 (1:200; Abcam Co., United Kingdom), anti-cleaved caspase-3 (1:500; Abcam Co., United Kingdom), or anti-β-actin (1:8,000; Santa Cruz Biotechnology). Secondary antibodies were horseradish peroxidase conjugated to mouse anti-rabbit/mouse immunoglobulin G (1:10,000; Sigma-Aldrich). Protein band densities were quantified using ImageJ software (NIH, Frederick, MD, United States) and were normalized to β-actin. Final data were expressed as a percent of that obtained from the control group.

### Reverse Transcription PCR

Six rats from each group were anesthetized and quickly decapitated 24 h after behavioral testing, and DG regions were carefully dissected on ice. Total RNA was isolated using the RNA rapid extraction kit (Aidlab, China) according to the manufacturers’ instructions. The total RNA was reverse transcribed into cDNA using the Revert Aid First Strand cDNA Synthesis kit according to the manufacturers’ instructions and subsequently amplified by PCR with specific primers (Supplementary Table 1). PCR products were separated by electrophoresis, and images were captured with the use of the Gel Image Analysis System (Bio-Rad, United States). Intensities of bands were analyzed using Image-Pro Plus 6.0 software, and values were normalized to GAPDH.

### Terminal Deoxynucleotidyl Transferase dUTP Nick-End Labeling (TUNEL) Staining

One day after behavioral tests, six rats from each group were anesthetized and transcardial perfused with 4% PFA. Brains were removed and fixed in PFA overnight at 4°C followed by a graded dehydration (10, 20, and 30% sucrose solution). Brain samples were cut into serial coronal frozen slices (30 μm) by oscillating blade microtome (Leica VT1200). Slices containing the DG regions (from bregma: AP, −3.20 mm ∼−3.60 mm; ML, ±0. mm; DV, −4.8 mm) were selected for TUNEL staining. Neuronal apoptosis in DG hippocampus was detected using TUNEL staining kit (KeyGen BioTech, China) according to the manufacturer’s protocol. Slides were counter-stained with DAPI. Images were captured using a scanning laser confocal microscope (LSM780; Carl Zeiss, Germany). Three randomly selected different fields (500 × 500 μm grids) of TUNEL-positive cells per section were selected, and calculations were performed within six pictures per rat. The number of active TUNEL and DAPI double-positive cells was calculated as the mean of the numbers obtained from six pictures from each rat. Counting was performed in a blinded manner.

### Statistical Analysis

The SPSS version 13.0 was used to analyze the data. All data were presented as the means ± SEM. Statistical significance of differences among groups was evaluated by one-way or two-way analysis of variance (ANOVA) followed by the Bonferroni’s test for multiple *post hoc* comparisons of means. A value of *p* < 0.05 was required for results to be considered as statistically significant.

## Results

### Fluoxetine Decreased Neuroinflammatory Responses in DG Hippocampus of Rats Exposed to CUMS

**FIGURE 1:**
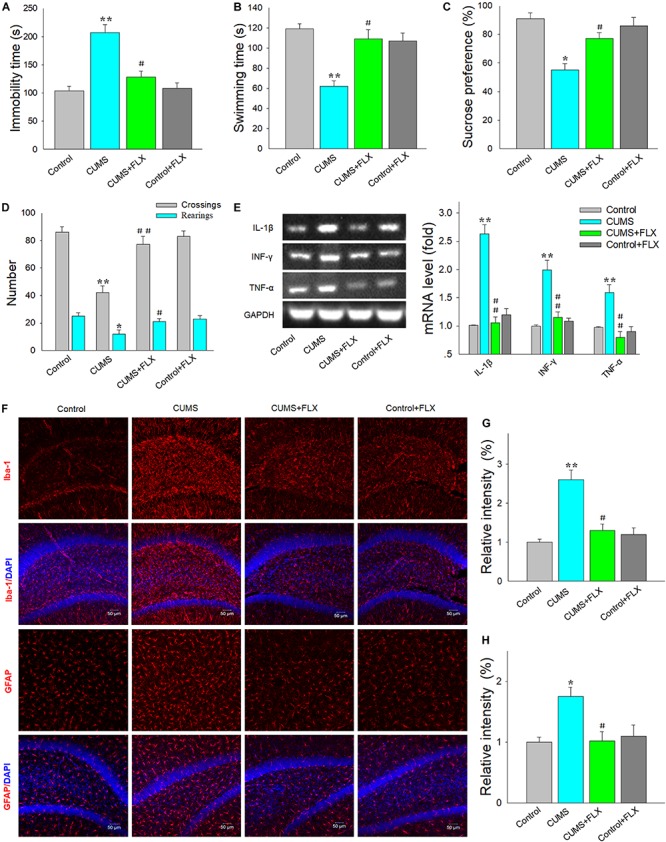
Fluoxetine suppresses glial activation and inflammatory cytokine expression resulting from chronic unpredictable mild stress (CUMS) exposure. **(A)** Fluoxetine reduced the increases in immobility times of CUMS rats in forced swim test. **(B)** Fluoxetine increased the decreases in swimming times of CUMS rats. **(C)** Fluoxetine reversed the decreases in consumption of sucrose solution in CUMS rats in sucrose preference test. **(D)** Fluoxetine reversed the decreases in exploratory activity of CUMS rats in open field test. **(E)** Reverse transcription (RT)-PCR assays of mRNA expression levels of interleukin-1β (IL-1β), tumor necrosis factor-α (TNF-α), and interferon gamma (IFN-γ) within dentate gyrus (DG). **(F)** Immunofluorescence staining revealed the Iba1-positive microglial cells within DG (AP, –3.25 mm from bregma) and anti-glial fibrillary acidic protein (GFAP)-positive astrocytes within DG (AP, –3.35 mm from bregma). Scale bar is 50 μm. **(G)** Bar graphs showing intensities of immunofluorescence signals of Iba1-positive microglial cells. **(H)** Bar graphs showing intensities of immunofluorescence signals of GFAP-positive astrocytes. For behavioral tests, *N* = 24 per group. For other experiments, *N* = 6 per group. *p < 0.05, **p < 0.01 compared to control group; ^#^*p* < 0.05, ^##^*p* < 0.01 compared to CUMS group (FLX, fluoxetine).

Results from the FST demonstrated that there was significant difference among the four groups regarding the immobility times [*F*(3,92) = 17.39, *p* < 0.05] and swimming times [*F*(3,92) = 15.78, *p* < 0.05]. *Post hoc* analysis revealed that daily treatment with fluoxetine for 2 weeks significantly decreased immobility times (*p* < 0.05) (Figure 1A) and increased swimming times (*p* < 0.05) (Figure 1B) in CUMS rats. In the SPT, the percent of sucrose consumption was significantly different among the four groups [*F*(3,92) = 15.16, *p* < 0.05]. *Post hoc* analysis revealed that fluoxetine treatment effectively increased the sucrose preference in rats exposed to CUMS (*p* < 0.05) (Figure 1C). The OFT showed that there was a significant difference among the four groups about the horizontal (crossings) [*F*(3,92) = 17.82, *p* < 0.05] and vertical (rearing) [*F*(3,92) = 16.91, *p* < 0.05] activities, while the fluoxetine treatment increased both of the horizontal (*p* < 0.05) and vertical (*p* < 0.05) activities in rats exposed to CUMS (Figure 1D). More importantly, there was a significant difference among the four groups regarding the expression of pro-inflammatory cytokine IL-1β [*F*(3,20) = 18.97, *p* < 0.05], interferon gamma (IFN-γ) [*F*(3,20) = 19.24, *p* < 0.05], and TNF-α [*F*(3,20) = 17.52, *p* < 0.05]. *Post hoc* analysis showed that fluoxetine treatment significantly attenuated the expression of pro-inflammatory cytokines (*p* < 0.01 respectively) (Figure 1E). Meanwhile, the activity levels of microglia [*F*(3,20) = 18.63, *p* < 0.05] and astroglia [*F*(3,92) = 18.63, *p* < 0.05] were significantly different among the four groups, while fluoxetine treatment suppressed the CUMS-induced microglia and astroglia activation within the DG region (*p* < 0.05 for both) (Figures 1F–H). Such findings indicate that fluoxetine produced a suppression of increases in inflammatory responses that may contribute to depression.

### Fluoxetine Decreased Neuronal Apoptosis in DG Hippocampus of Rats Exposed to CUMS

**FIGURE 2:**
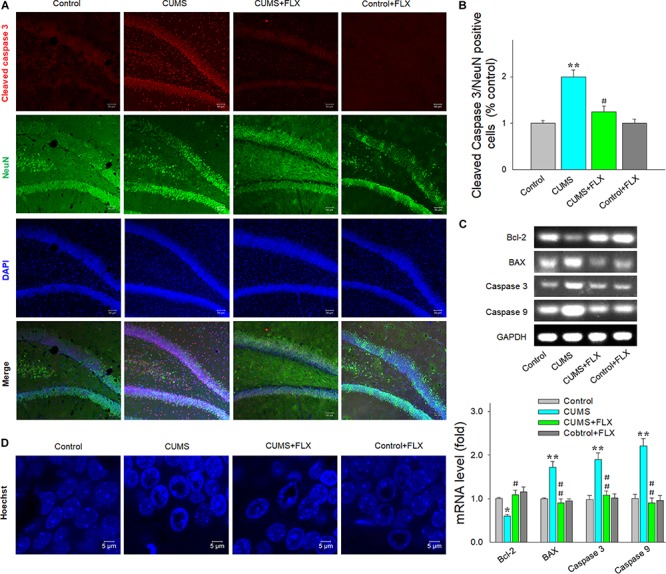
Fluoxetine decreases neural apoptosis in hippocampal DG resulting from CUMS exposure. **(A)** Immunofluorescence staining revealed double-labeled cleaved caspase 3/NeuN-positive cells within DG (AP, –3.40 mm from bregma). Scale bar is 50 μm. **(B)** Bar graphs showing that fluoxetine significantly decreased the number of double-labeled-positive cells in CUMS rats. **(C)** RT-PCR assays of mRNA levels of Bcl-2, Bax, cleaved caspase 3 and caspase 9 within DG. **(D)** Hoechst-33258 staining revealing the morphological changes of nuclei. Scale bar is 5 μm. *N* = 6 per group. *p < 0.05, **p < 0.01 compared to the control group; ^#^*p* < 0.05, ^##^*p* < 0.01 compared to CUMS group (FLX, fluoxetine).

In the DG regions of the hippocampus, neuronal death was clearly observed after 5 weeks of CUMS exposure as revealed with immunofluorescence staining. The density of positive cleaved caspase 3 and NeuN double-labeled cells showed a significant difference among the four groups [*F*(3,20) = 16.15, *p* < 0.05]. *Post hoc* analysis revealed that fluoxetine treatment reversed the increased density of positive cleaved caspase 3 and NeuN double-labeled cells caused by CUMS (*p* < 0.05) (Figures 2A,B). In addition, mRNA levels of the apoptosis-related proteins Bcl2 [*F*(3,20) = 12.96, *p* < 0.05], Bax [*F*(3,20) = 15.67, *p* < 0.05], caspase 3 [*F*(3,20) = 16.21, *p* < 0.05], and caspase 9 [*F*(3,20) = 17.47, *p* < 0.05] were all significantly different among the four groups, while fluoxetine treatment reversed the increased levels of Bax, caspase 3, and caspase 9 within the DG region of rats exposed to CUMS (*p* < 0.01 respectively), effects which were accompanied with the elevation of the decreased expression levels of Bcl-2 (*p* < 0.05) (Figure 2C). Moreover, Hoechst-33258 staining showed that CUMS rats exhibited nuclear chromatin margination, aggregation, and condensation, typical changes of apoptotic nuclei (Figure 2D). In contrast, these apoptotic morphological changes and overexpression of pro-apoptotic factors were significantly decreased in response to fluoxetine treatment.

### The p38 Is Involved in the Chronic Stress-Induced Neural Apoptosis and Depressive Behaviors of CUMS Rats

**FIGURE 3:**
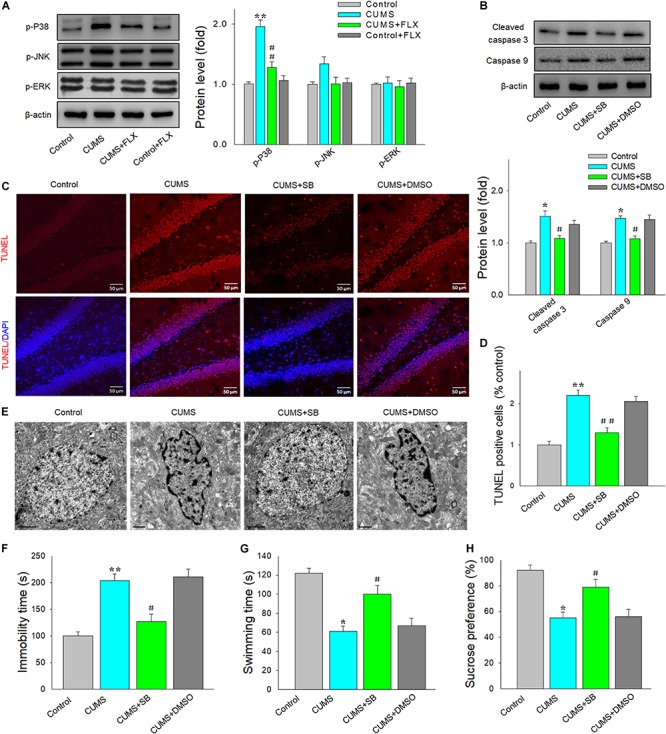
Blocking of the p38 mitogen-activated protein kinase (MAPK) pathway reduces neuroinflammatory responses resulting from CUMS exposure. **(A)** Western blotting was used to detect the main MAPK family members in DG. **(B)** SB203580 decreased expression levels of cleaved caspase 3 and caspase 9 in CUMS rats. **(C)** SB203580 decreased the number of TUNEL-positive cells in DG (AP, –3.50 mm from bregma) of CUMS rats. Scale bar is 20 μm. **(D)** Bar graphs showing significant differences in number of TUNEL-positive cells among the four groups. **(E)** SB203580 attenuated morphological changes in nuclei within DG neurons in CUMS rats. Scale bar is 1 mm. **(F)** SB203580 reduced immobility times in CUMS rats. **(G)** SB203580 increased swimming times of CUMS rats. **(H)** SB203580 increased sucrose solution consumption in CUMS rats. *N* = 6 per group. *p < 0.05, **p < 0.01 compared to the control group; ^#^*p* < 0.05, ^##^*p* < 0.01 compared to the CUMS group (FLX, fluoxetine; SB, SB203580).

To investigate the possible mechanisms underlying the anti-inflammatory and anti-apoptotic effects resulting from fluoxetine, western blotting was used to detect the crucial members of the MAPK family, which participate in a signaling cascade controlling cellular responses to cytokines and stress. We found that the phosphorylation levels of p38 MAPK were significantly different among the four groups [*F*(3,20) = 16.82, *p* < 0.05]. Interestingly, *post hoc* analysis showed that fluoxetine treatment significantly reversed this increase in p38 phosphorylation levels within the DG region in response to CUMS exposure (*p* < 0.05) (Figure 3A). However, there were no significant changes were observed with regard to phosphorylation levels of JNK and ERK (*p* > 0.05 for both). To corroborate these findings, we next injected SB203580 to block p38 MAPK activity prior to daily CUMS exposure. The results showed that the protein levels of cleaved caspase 3 [*F*(3,20) = 12.88, *p* < 0.05] and caspase 9 [*F*(3,20) = 13.92, *p* < 0.05] within DG regions were significantly different among the four groups, while SB203580 reversed the increased levels of these two apoptosis-related proteins in rats exposed to CUMS (*p* < 0.05 for both) (Figure 3B). In addition, SB203580 pretreatment effectively reduced the increased number of TUNEL-positive apoptotic cells within DG regions caused by CUMS exposure (*p* < 0.01) (Figures 3C,D), as well as attenuated the morphological changes in apoptotic nuclei, including nuclear chromatin margination, aggregation, and condensation (Figure 3E). Finally, behavioral results showed that SB203580 significantly reversed the increased immobility (*p* < 0.05), decreased swimming times (*p* < 0.05) in the FST (Figures 3F,G), and the decreased sucrose consumption that result from CUMS exposure (*p* < 0.05) (Figure 3H).

### Fluoxetine Ameliorated p38-Induced Neural Injury and Depressive Behaviors in Rats

**FIGURE 4:**
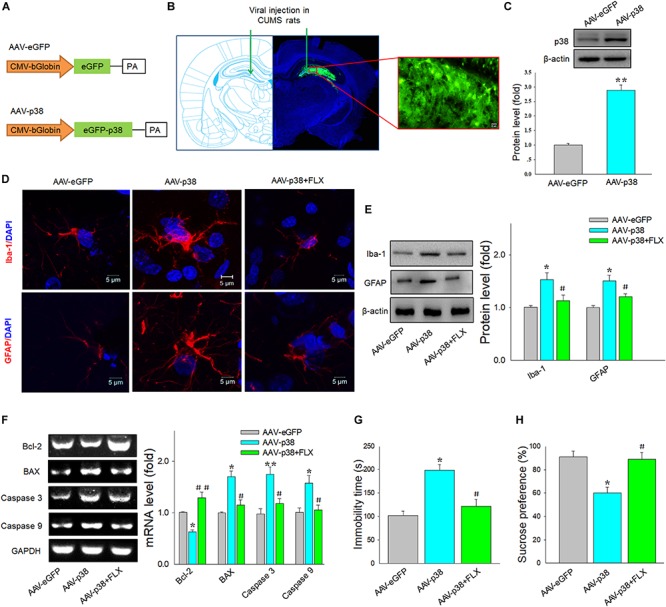
Fluoxetine ameliorated neuronal injury and behavioral changes resulting from up-regulation of p38 in DG. **(A)** Schematics of AAV vectors engineered to overexpress p38 or eGFP control. **(B)** Illustration of bilateral viral injection of AAV–p38 in DG. **(C)** Validation of the efficient overexpression of p38 in DG. **(D)** Fluoxetine prevented the morphological changes in microglia and astrocytes within the DG (AP, –3.35 mm from bregma) caused by p38 overexpression. **(E)** Fluoxetine reduced Iba1 and GFAP levels in DG. **(F)** Fluoxetine reduced the increased levels of pro-apoptotic factors caused by p38 overexpression in DG. **(G)** Fluoxetine reduced immobility times. **(H)** Fluoxetine increased sucrose solution consumption. For behavioral tests, *N* = 18 per group. For other experiments, *N* = 6 per group. *p < 0.05, **p < 0.01 compared to the control group; ^#^*p* < 0.05, ^##^*p* < 0.01 compared to the CUMS group.

To further confirm whether the p38 signaling pathway is involved in the neuroprotective and antidepressant effects of fluoxetine, the AAV–p38 virus was constructed (Figure 4A) and bilaterally infused into DG regions of rats to overexpress p38 (Figure 4B). Results showed that the expression level of p38 protein was significantly increased after AAV–p38 virus injection (*p* < 0.01) (Figure 4C). Immunofluorescence assays revealed that fluoxetine significantly ameliorated the rounded, activated-like appearance of microglia and cellular hypertrophy of astrocytes induced by p38 overexpression (Figure 4D). Moreover, the protein levels of Iba-1 [*F*(2,15) = 12.84, *p* < 0.05] and GFAP [*F*(2,15) = 14.16, *p* < 0.05] in the DG region were significantly different among the three groups, while fluoxetine reduced the increased levels of Iba-1 and GFAP induced by p38 overexpression (*p* < 0.05 for both) (Figure 4E). Moreover, up-regulation of p38 elevated the mRNA levels of pro-apoptotic factors Bax (*p* < 0.05), caspase 3 (*p* < 0.05), and caspase 9 (*p* < 0.05), which all suppressed by fluoxetine treatment (*p* < 0.05) (Figure 4F). Finally, behavioral results showed that the immobility times [*F*(2,51) = 14.76, *p* < 0.05] and sucrose consumption [*F*(2,51) = 15.02, *p* < 0.05] were significantly different among the three groups, while fluoxetine also significantly ameliorated the depression-like behaviors in these rats (*p* < 0.05 for both) (Figures 4G,H).

## Discussion

Fluoxetine has been used as an antidepressant on the basis of its capacity to inhibit serotonin reuptake in depressed patients (Perlis et al., 2004). Nevertheless, it remains unclear whether this represents the sole mechanism through which fluoxetine exerts this antidepressant effect. If additional mechanisms exist, it may be possible to develop novel therapeutic targets in the treatment of depression. In the present study, we demonstrate that fluoxetine exerts neuroprotective effects against neural injury in a CUMS rat model of depression. These neuroprotective effects of fluoxetine, in part, involve alleviating the neuroinflammation and neuronal apoptosis resulting from CUMS exposure via suppression of the p38 MAPK pathway. Our findings that specific inhibition of p38 reduces neural injury in the DG of the hippocampus as well as ameliorates depressive behaviors suggest that the p38 pathway could serve as a potential target in the treatment of depression.

It has been demonstrated that brain inflammation represents the single most critical pathophysiological risk factor in the genesis of depression (Haapakoski et al., 2015; Qin et al., 2017). This enhanced neuroinflammation can then induce neuronal apoptosis, which is believed to contribute to the neuronal deterioration observed in depression (Villas Boas et al., 2019). Although inflammation-induced apoptosis is the most important process leading to neuronal dysfunction in the progression of depression, the underlying pathophysiological mechanisms have not been well understood. Interestingly, the antidepressant, fluoxetine, has been shown to exert significant neuroprotective effects in many neurological disorders, including ischemic stroke patients (Chollet et al., 2014), animal models of stroke (Singh and Chopra, 2014), and several animal models of neurodegenerative diseases, such as Parkinson’s disease (Kohl et al., 2012) and Alzheimer’s disease (Qiao et al., 2016). These results reveal that the antidepressant mechanisms of fluoxetine might be complicated in depression treatment.

Within the central nervous system (CNS), microglia represent the primary resident immune cells responsible for responding to various neuropathological stimuli, including stress, injury, and infection (Dheen et al., 2007). Activation of these microglia results in the rapid proliferation of cellular densities and hypertrophy, followed by activation of astrocytes and the release of pro-inflammatory cytokines, including IL-1β, TNF-α, and IFN-γ. This cascade can then result in neuronal damage and cell death, which is often associated with the duration or severity of the mood disorder in patients with MDD (Miller et al., 2009). In the present study, we demonstrate that fluoxetine reduced pro-inflammatory cytokine levels, as well as microglial and astrocytic activation within the DG hippocampus. These results suggest that an anti-inflammatory role may contribute to fluoxetine’s ability to prevent depression-like behaviors following CUMS exposure. Pro-inflammatory cytokines are now considered as important pro-apoptotic factors involved in the pathological process of neurological disorders (Griffin et al., 2018), and accumulating evidence has been accrued demonstrating that IL-1β is a key contributor to the inflammation-induced neuronal death in neurological diseases (Kaufmann et al., 2017; Tsai, 2017). Here, we found that fluoxetine reduced NeuN and cleaved caspase 3 double-labeled cells in rats exposed to CUMS, which substantiated that neuronal cells were undergoing apoptosis after chronic stress. Fluoxetine further downregulates the pro-apoptotic factors Bax, caspase 3, and caspase 9, and an accompanying upregulation of Bcl-2. These results suggest that neuroinflammatory responses, which can result in cell death within an animal model of depression as induced by chronic stress, could be effectively reversed with fluoxetine treatment.

All of the evidence as presented above leads to the proposal that the overexpression of pro-inflammatory cytokines may activate one potential pathway through which neuroinflammatory mechanisms may proceed to cause neuronal apoptosis, eventually leading to depression. Results from a previous study have indicated that IL-1β could result in synaptic deficits and axon developmental disorders through activation of the p38 MAPK signaling pathway in septic neonatal rats (Han et al., 2017). Moreover, it has also been reported that p38 MAPK is crucial for caspase 3 activation and, thus, induces neuronal cell apoptosis in the cerebral ischemia reperfusion injury model (Kim et al., 2015; Li and Ai, 2017). These results suggest that the p38 MAPK pathway appears to serve as a bridge between neuroinflammation and neuronal apoptosis, and thus promotes depression-like behaviors in this CUMS-induced rat model of depression. Our present results demonstrate that CUMS exposure mainly triggers the phosphorylation of p38, as opposed to ERK and JNK, and fluoxetine significantly inhibits the activation of p38, but fails to influence that of ERK and JNK. These results suggest a relative specificity of the p38 pathway in this process. Therefore, to further substantiate the potential crosstalk roles of p38 in the pathogenesis of this depression model, we first blocked p38 MAPK activity with the use of its specific inhibitor, SB203580, during CUMS exposure. SB203580 treatment significantly suppressed apoptosis in DG, as well as rescues the depressive phenotypes in rats induced by chronic stress. In addition, fluoxetine ameliorated the pathophysiological changes caused by overexpression of p38 in DG regions via infusion of a constructed AAV–p38. Taken together, these results indicate that the p38 pathway markedly contributes to the pathogenesis of depression and, fluoxetine, in part, exerts its neuroprotective effects by downregulating this p38 pathway. However, the detailed molecular and clinical mechanisms of how fluoxetine regulates p38 signaling needs further investigation.

## Conclusion

In summary, the present study revealed a novel neuroprotective mechanism whereby fluoxetine exerts antidepressant effects via preventing neural inflammation and apoptosis by inhibiting the p38 MAPK signaling pathway in a rat model of depression. The identification of this pathway suggests that it may provide an important potential target for the development and use of novel antidepressants to treat depressive symptoms.

## Data Availability Statement

The raw data supporting the conclusions of this article will be made available by the corresponding author upon reasonable request.

## Ethics Statement

The animal study was reviewed and approved by the guidelines of the Ethics Committee of the Medical Department of Nanchang University and the International Guiding Principles for Animal Research provided by the International Organizations of Medical Sciences Council (CIOMS).

## Author Contributions

YZ and JL contributed to the study design and analyses of the data. YZ and PS performed the biochemical analysis and drug injections, immunohistochemistry, and confocal imaging analysis. MW and MX performed depression model and behavioral tests. JL wrote the first draft and YZ participated in the subsequent drafts.

## Conflict of Interest

The authors declare that the research was conducted in the absence of any commercial or financial relationships that could be construed as a potential conflict of interest.
