# Supplementary figures and images for: Neuroprotective Effects of Fluoxetine Against Chronic Stress-Induced Neural Inflammation and Apoptosis: Involvement of the p38 Activity
Source: Front Physiol. 2020 May 11;11:351. doi: 10.3389/fphys.2020.00351 (PMC7233199; doi:10.3389/fphys.2020.00351)

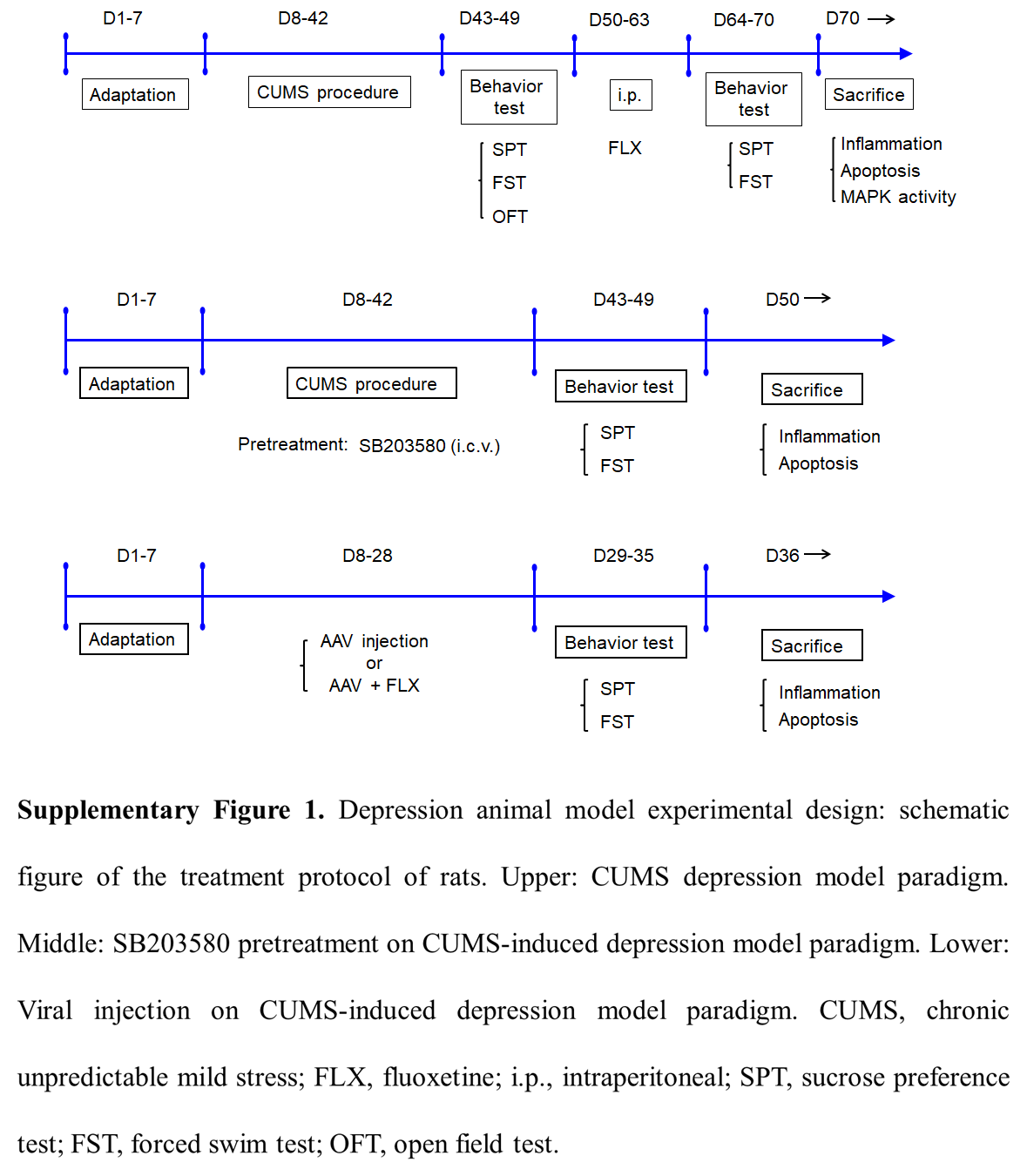

Supplement: Supplementary file 1 [file Image_1.tif]
