# Supplementary material for: Neuroprotective Effects of Fluoxetine Against Chronic Stress-Induced Neural Inflammation and Apoptosis: Involvement of the p38 Activity
Source: Front Physiol. 2020 May 11;11:351. doi: 10.3389/fphys.2020.00351 (PMC7233199; doi:10.3389/fphys.2020.00351)
Supplement: Supplementary file 3 [file Data_Sheet_1.docx]

Research Highlights

1. Fluoxetine ameliorated depression-like behaviors in CUMS-exposed rats.
2. Fluoxetine suppressed neural inflammation and apoptosis in DG hippocampus of depressed rats.
3. Fluoxetine prevented the activation of p38 MAPK caused by CUMS exposure.
4. Fluoxetine protects against neural injury and depressive behaviors in rats via suppression of p38.
5. Blocking of p38 pathway is a potential therapeutic avenue for depression treatment.
